# The Physiology of Hypnotism

**Published:** 1894-11

**Authors:** Alfred H. Porter

**Affiliations:** Philadelphia


### THE PHYSIOLOGY OF HYPNOTISM.[1]

BY ALFRED H. PORTER, D.D.S., PHILADELPHIA.

THE study of hypnosis differentiates two states, the auto-hypnotic or self-induced condition, and that effected by the more potent energies of the hypnotist in their irradiation antagonizing those of the patient. In regard to the latter, physicists have scarcely dared to conceive of such a mode of energy acting through space, much less have they attempted to subject it to experiment. The general willingness to explore the field now manifested by experimental scientists is due to the establishment of the marvellous essentially dynamic hypnotic phenomena on a firm basis without their aid; and to a well-founded belief that critical analysis must lead to the illustration and development of natural law itself.

The most natural explanation of the radiant energy of the hypnotist, since under certain conditions the tissue manifests its electrical currents, is that it is a type of electrical radiation.

Although Galvani's experiment on animal electricity in 1786 founded the ever-widening field of voltaic electricity, and led to modern electrotonic physiology, no demonstration has been made that the conditions required for the evident manifestation of electrical separation in the phenomena of electrotonus are analogous to the special physiological conditions required for the concentration of energies characteristic of both hypnotist and patient. This is amazing, for it is in spite of the fact that the more observant hypnotists incontestably have a sense of the efflux of energy, and the patients of its absorption.

Many may not be conscious of this subtle sense of effort and

---

[1] Read before the Alumni Association of the Department of Dentistry, University of Pennsylvania, June, 1894.

impact, but even in normal life few have any conception of a muscular sense, and the far more important nervous sense concomitant with the discharge of energy in the nervous centres that constitutes the basis of every form of conscious and unconscious mental activity.

Leaving the protestation of the hypnotists of a subjective sensation out of view, the objective phenomena were striking enough to attract attention. Popularly the energy exerted by the hypnotist was termed "animal magnetism." Such designation and the fact that to magnets vicarious powers could be delegated apparently by the hypnotist or "magnetizer" led good physiologists astray. Because dummy magnets produced the same effect on the patient as the "hypnotized magnets," the idea that magnetism could explain the transfer of energy fell undeservedly into disrepute.

In many of its phases magnetism manifests essentially electrical phenomena, leading many to regard it as a mode of electricity; and it cannot be neglected in discussing the transformation of the chemical energies of the tissues into electrical energy in normal physiological states or the abnormal states of hypnosis, for Dewar has proved that under extreme pressure oxygen is strongly magnetic; and, moreover, the energies required for the association and disassociation of the gases of the blood form the *rationale* primarily of the respiratory and circulatory activities, and, ultimately, of the whole corporeal energies, visible and invisible. These electrical energies are initiated by the magnetism of the iron of the oxy-hæmoglobin of the blood attracting the oxygen. Further research would have led the experimenters to find that the mere perception of the dummy magnet had completely hypnotized the patient, and may be long before the school of Nancy to strike the key-note of hypnosis,—the potency of suggestion.

It is well to remember that the electrical discharge and the phenomena of attraction and repulsion are forms of one all-powerful irradiative agency. The most comprehensive idea of electric action is to imagine that in one place opposing energies have condensed or coiled up the all-pervading ether. The release of this would mean the propagation of waves of all amplitudes, intensities, alternations, in all directions, forming central nodes of rest and surrounding vortices. The coincidence of crests with troughs would form places of absolute destruction of motion, enabling us to conceive of electricity as being vibratory, instead of an uninterrupted stream of energy.

This extensive idea of the operation of electricity is supported by epoch-making discoveries of the last few years by Hertz, of Vienna. Electrical currents no more fairly represent the possibilities of electricity than a river represents the opposing seas and maelstroms of Cape Horn.

Let us apply this expansive idea of coil and recoil of the ether denoting the electrical discharge to animal electricity, remembering that a current of electricity is the concentration of energies otherwise diffused, kept in one direction by the wire, just as the narrow bay of Fundy concentrates the tides of the Atlantic Ocean, or a speaking-tube concentrates the ample air vibrations. We will begin with Galvani's time-worn experiment, where the muscle contracts in the neighborhood of an electrical discharge. This discharge is obtained by the contact of two heterogeneous metals. The surface particles of the metals are vortices of different potentials; they will, as it were, unwind the energies of each other, producing a disturbance of the ether, but the ether-waves, although measurable by physical apparatus, are too long to be noted by the eye or ear, much less the grosser forms of tissue that form the nerves and muscles of the limbs. The fact is, the muscle and nerve will not respond to such waves. In physical language, the nerve-energies will not resonate to such long feeble waves. By his wire Galvani condensed them into a current, the response of the nerve-cells was immediate, their energies which maintained the natural tonus of the nerve and muscle were inhibited, and the contraction ensued. This contraction, like the idio-muscular, spasmodic, tetanic, rigor-mortis and all pathological forms, indicates functional disintegration of controlling nervous centres, and is precisely the hypnotic state of energies.

The prime cause of the waves was, as we have said, the discharge of energy consequent on the contiguity of two heterogeneous metals. The question at stake is, Are the heterogeneous energies of hypnotist and patient analogous to those of the metal? and are their contiguity and mutual attention equivalent to closing a circuit with a wire? Moreover, do the energies of the patient succumb to those of the hypnotist because of lesser momentum and negative character?

The phenomena of nerve and muscle electrotonus are easily apprehended with this idea of the diffusive, undulatory, vocative action of electricity. The anelectrotonic condition in which the constant current or concentration of energy increases the natural energies is the condition of the hypnotist. The kata-electrotonic condition, where the same energies are inhibited but the impressibility increased, is that of the patient. Let us consider that the anelectrotonic and kata-electrotonic activities are manifested in the same nerve or muscle and by the same constant current. A similar opposite condition prevails between hypnotist and patient, who both concentrate their energies.

The design of this paper is to elucidate, however, the auto-hypnotic condition, as to which no speculation is needed, standing on an unassailable foundation, and accounted for rationally by normal physiological operation and the abnormal pathological states of dementia.

The condition goes as far back as history records; indeed, it seems to have formed the basis of ancient mythology and medicine. As in chemical anæsthesia, the operator is an adjunct, but with powers far transcending the administrator of the narcotic, for the sole reason that the patient is amenable to suggestion.

A disk is placed before the patient, the continual perception of which induces hypnosis.

The simple condition of undivided attention is fulfilled in many ancient and modern religions, producing essentially the hypnotic state. The idolater, gazing reverently on the face of the idol, the more rapt his gaze, saw a halo of glory surround the head apparently. The continuous incantation of the priests was the similar continuous stimulation of another sense-organ, and accelerated his reverie. There was certainly no super-natural efflux of energy from the idol, but there was the natural efflux or rebound of invisible waves of energy termed light, as there is from every object of vision. Were it not for this irradiation and various intensities, amplitudes, and alternations of the waves, no idea of any color, size, or configuration of objects would be obtained. After all, they are our old friends, the electrical waves,—precisely speaking, electro-magnetic waves,—more intense and of shorter wave-length, just long enough to cause the response of the myriads of rods and cones with which the retina is studded.

We must understand that the very invisibility of the waves enables us to perceive any object, for, to give us an idea of a large object, many crests must coincide to form a wave of large amplitude and correspondingly affect the retina. If the amplitudes of the waves increase, as they often do from mist or other physical causes, the object would appear larger.

Indeed, as the sun declines, the approaching uniform hue of the landscape and the loss of detail of configuration is simply due to a coinciding of the wave-lengths and amplitudes.

The physiological text-books tell us that an image exists on the retina, and in some inscrutable way the brain transmutes the image into perception, and, in a similar way, transmutes the waves of energy that traverse the auditory nerve into sound. This is essentially misleading, and has hindered an accurate conception of the nervous energies of the brain.

Retina, optic nerve, and central brain cell in the optical centre are anatomically continuous and they are physiologically continuous. The electrical vibratory image exists on the retina, optic nerve, and central cell, in one specific phase of energy. Each contributes impartially its share for the energy of perception. But apart from this identical action each has a specific function in relation to the environment. The retina responds to the million phases of energy forming the visual world; its function is to respond to many of them simultaneously, for which purpose its nervous layers admirably adapt it. The function of the optic nerve is to convey these vibratory pictures just as wires through electric stress are made to carry them in the physical laboratory, but the supremacy of the brain lies in the fact that it retains them. Yet that retentive power is not a mode of perception. The restimulation of the cell forms the basis of memory, its response to a like phase of energy from the optic nerve is the basis of recognition; the interaction and expansion of the energies of the cerebral cells form the basis of deliberation; their manifestation of kinetic energy is the basis of the tonicity of the centres, and effects the restraint of their mechanical action characteristic of the normal man.

We shall endeavor to demonstrate that the continued stimulus of the retina, tympanum, or any sensory apparatus leads to the removal of this inhibition, and leaves each cell open to the stimulus of suggestion without power to antagonize it,—the physiological *rationale* of the hypnotic state.

(To be continued.)

